# Key Steps in Developing a Cognitive Vaccine against Traumatic Flashbacks: Visuospatial Tetris versus Verbal Pub Quiz

**DOI:** 10.1371/journal.pone.0013706

**Published:** 2010-11-10

**Authors:** Emily A. Holmes, Ella L. James, Emma J. Kilford, Catherine Deeprose

**Affiliations:** Department of Psychiatry, University of Oxford, Oxford, United Kingdom; Chiba University Center for Forensic Mental Health, Japan

## Abstract

**Background:**

Flashbacks (intrusive memories of a traumatic event) are the hallmark feature of Post Traumatic Stress Disorder, however preventative interventions are lacking. Tetris may offer a ‘cognitive vaccine’ [1] against flashback development after trauma exposure. We previously reported that playing the computer game Tetris soon after viewing traumatic material reduced flashbacks compared to no-task [1]. However, two criticisms need to be addressed for clinical translation: (1) Would all games have this effect via distraction/enjoyment, or might some games even be harmful? (2) Would effects be found if administered several hours post-trauma? Accordingly, we tested Tetris versus an alternative computer game – Pub Quiz – which we hypothesized not to be helpful (Experiments 1 and 2), and extended the intervention interval to 4 hours (Experiment 2).

**Methodology/Principal Findings:**

The trauma film paradigm was used as an experimental analog for flashback development in healthy volunteers. In both experiments, participants viewed traumatic film footage of death and injury before completing one of the following: (1) no-task control condition (2) Tetris or (3) Pub Quiz. Flashbacks were monitored for 1 week. **Experiment 1**: 30 min after the traumatic film, playing Tetris led to a significant reduction in flashbacks compared to no-task control, whereas Pub Quiz led to a significant increase in flashbacks. **Experiment 2**: 4 hours post-film, playing Tetris led to a significant reduction in flashbacks compared to no-task control, whereas Pub Quiz did not.

**Conclusions/Significance:**

First, computer games can have differential effects post-trauma, as predicted by a cognitive science formulation of trauma memory. In both Experiments, playing Tetris post-trauma film reduced flashbacks. Pub Quiz did not have this effect, even increasing flashbacks in Experiment 1. Thus not all computer games are beneficial or merely distracting post-trauma - some may be harmful. Second, the beneficial effects of Tetris are retained at 4 hours post-trauma. Clinically, this delivers a feasible time-window to administer a post-trauma “cognitive vaccine”.

## Introduction

### Trauma and Posttraumatic Stress Disorder

The psychological impact of trauma is a major challenge to human health worldwide. The majority of healthy individuals are liable to suffer a traumatic event at some point in their life time [2], and are thus placed at risk of developing Posttraumatic Stress Disorder (PTSD) [3]. After experiencing a traumatic event, people can suffer from disturbing intrusive memories of the event, commonly referred to as flashbacks, in which the traumatic material comes back to mind as unwanted images and scenes of the trauma. These involuntary trauma memories are associated with significant emotion and distress [4], [5]. At their worst, such flashbacks to trauma can persist for extended periods of time, causing significant distress and impairment. Re-experiencing symptoms such as flashbacks are the hallmark characteristic of PTSD [6] and a precursor to the disorder [7]. While we have successful treatments for patients who have developed full blown PTSD [e.g. Cognitive Behavioural Therapy; 8] these treatments can only be administered after 1-month post-trauma (once the disorder is diagnosed). We do not yet have evidence-based methods to *prevent* the build up of symptoms. That is, we lack early interventions to treat people in the aftermath of trauma exposure [8]. In particular, we now know there are substantial international at-risk populations for PTSD such as soldiers involved in combat [9]. Further, the scale of trauma world-wide is significant including war, terrorism, natural disasters, interpersonal violence and so forth [10], [11], [12]. Thus, the trauma field is in critical need of easily accessible treatment innovations to reduce PTSD symptoms in the immediate aftermath of trauma.

### A ‘cognitive vaccine’ against flashbacks via visuospatial computer games such as Tetris: a treatment rationale from cognitive science

We proposed the development of computerized, low intensity, intervention against PTSD flashbacks for use as a preventative mental health strategy [1]. Specifically, we proposed that playing computer games such as Tetris post-trauma may offer a *‘cognitive vaccine’ to* inoculate against the build-up of flashbacks. This proposal was theory-driven from a cognitive neuroscience account of the sensory nature of trauma memory and interference with flashback formation. However, the same theory suggests other types of intervention may be harmful. Our theory postulates that:

1. Human memory differentiates visual and verbal components [13], [14], [15]
2. Pathological trauma flashbacks consist of sensory, visual images (i.e. vivid visual memories such as the sight of the blood-spattered body of a fellow soldier) [5], [6], [16]
3. Cognitive science shows that visuospatial cognitive tasks compete for resources with visual images [17], [18], [19], [20]
4. The biology of memory consolidation suggests a 6 hour time frame post-trauma within which memories are malleable [21]
5. Thus, visuospatial cognitive tasks given within 6 hours post-trauma will interfere with visual flashback memory consolidation, and reduce later flashbacks, as demonstrated in our previous study [1]
6. In contrast, verbal tasks post-trauma will not reduce flashbacks as verbal tasks compete with verbal, conceptual processing of the event but not the visual images that make up flashbacks [22], [23]
7. Further, verbal tasks post-trauma will compete with the type of verbal-conceptual processing necessary to make sense of what has happened and from clinical models may serve to increase (rather then reduce) later trauma flashbacks [24], [25], [26].

### Developing a safe cognitive intervention post-trauma: what are the next steps?

With regards to points 1–5 above, our previous paper in PLoS ONE [1] provided the first step in developing an intervention, demonstrating under experimental conditions that intrusive flashback memories were reduced by the visuospatial task Tetris [27], [28], [29]. Tetris [30] is a popular and widely-available computer game which requires mental rotation of stimuli, thus demanding on the cognitive neuropsychological domain of visuospatial working memory. However, critical questions remain:

(2) Would all computer games have the effect of reducing flashbacks via distraction/enjoyment, or might some games even be harmful?

A critical limitation of our 2009 study [1] is that there were only two experimental conditions (Tetris vs. no-task control), and we did not use another computer game as a comparison. It could be argued that Tetris may merely have distracted participants from focusing attention on the traumatic material, thereby reducing their flashbacks. Such an argument would suggest that any computer game might have a beneficial effect on flashbacks by being both enjoyable and distracting.

Interestingly, a counter argument to a “distraction account” is that according to the theory from cognitive science outlined in points 6–7 above, a comparison task such as a computer game requiring verbal processing should not only be ineffective but may even be harmful in increasing the development of later flashbacks. “Pub Quiz” [31]- a general knowledge, verbal computer game was used to specifically test these opposing hypotheses.

(1) Would the beneficial effects of playing Tetris on flashbacks be sustained if administered several hours post-trauma?

A second limitation of our 2009 study was that we only tested the intervention soon after trauma, whereas in the real world people may not have access to an intervention for several hours. Thus in Experiment 2, we considerably extended the time frame from 30 min to 4 hours post trauma to explore the impact of playing the computer games after a significant interval on the development of flashbacks.

In summary, we predicted that:

Experiment 1: playing the computer game Tetris 30 min after a traumatic film would significantly *reduce* flashbacks over 1-week compared to no-task control. Conversely, we predicted that playing the computer game Pub Quiz would significantly *increase* flashbacks compared to no-task control. In contrast to the impact on involuntary memory (flashbacks), deliberate memory for the traumatic events would be equivalent across conditions.

Experiment 2: effects would be maintained when the games were played 4 hours post-trauma.

## Results

### Experiment 1

**Figure 1:**
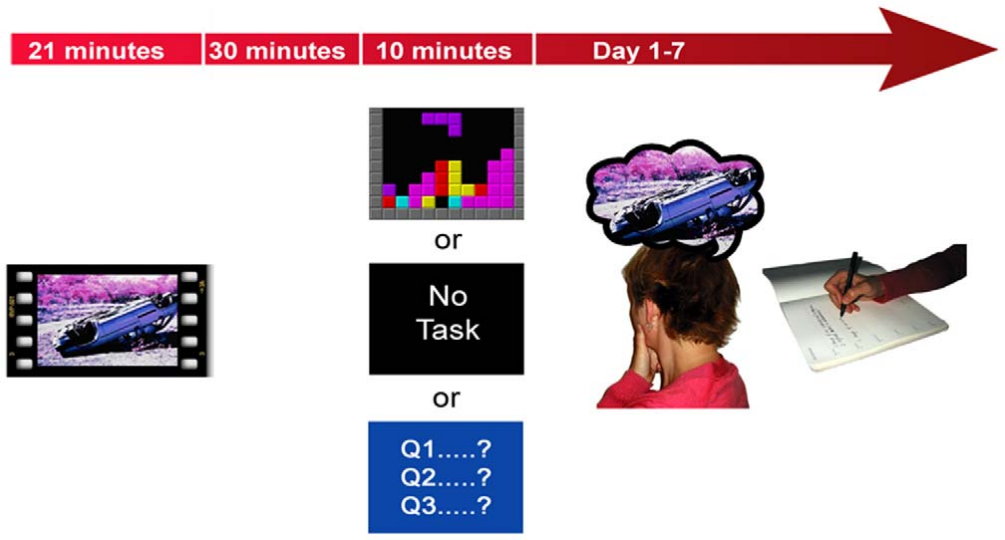
Experiment 1 study design overview. Participants completed the trauma film paradigm, a well established experimental analog for PTSD. All participants viewed a traumatic film followed by a 30-min structured break. Participants were then allocated to one of three experimental conditions [Tetris vs. no-task control vs. Pub Quiz] which they completed for 10 min. Afterwards participants in the computer game conditions rated their enjoyment of the game. Flashbacks (involuntary memories) were monitored for 1 week using an intrusion diary. After 1 week, diary compliance was checked and a test of voluntary memory (recognition memory test) for the trauma film was administered.

Sixty non-clinical participants watched a film of traumatic scenes of injury and death (n = 20 per group). Film viewing was followed by a 30-min structured interval before random assignment to one of three experimental conditions (figure 1). There were no baseline differences between the three groups in terms of age, number of previous traumatic events, depressive symptoms or trait anxiety (Table 1). Mood was equivalent between conditions pre-film, and as predicted, all conditions experienced comparable mood deterioration post-film (Table 2).

**Table 1:** Experiment 1 means and statistics for age and baseline assessments indicating experimental conditions did not differ.

Measure	No-task (n = 20)		Tetris (n = 20)		Pub Quiz (n = 20)		ANOVA
	mean	sem	mean	sem	mean	sem	
Age	29.05	2.68	25.90	2.11	26.75	2.24	*F*_(2,57)_ = 0.47 (NS)
Beck Depression Inventory (BDI)	5.35	0.91	4.90	1.04	5.85	1.03	*F*_(2,57)_ = 0.22 (NS)
Trait Anxiety (STAI-T)	37.20	1.98	39.10	1.95	36.60	2.10	*F*_(2,57)_ = 0.41 (NS)
Traumatic Experiences Questionnaire (TEQ)	1.60	0.39	1.50	0.22	1.90	0.47	*F*_(2,57)_ = 0.27 (NS)

**Table 2:** Experiment 1 means and statistics for mood assessment (pre vs. post-trauma film) indicating equivalent deterioration in mood over the film across conditions.

	No-task (n = 20)		Tetris (n = 20)		Pub Quiz (n = 20)		ANOVA		
	mean	sem	mean	sem	mean	sem	Time	Group	Group* Time
Pre-film mood	2.61	0.58	2.98	0.52	2.76	0.58	*F*_(2, 57)_ = 142.69§	*F*_(2, 57)_ = 0.20(NS)	*F*_(2, 57)_ = 0.68(NS)
Post-film mood	12.60	1.14	10.93	1.40	11.25	1.43			

§ p<0.01.

Following the 30-min structured interval period in which participants completed standardized filler tasks, a brief reminder task for the trauma film (neutral static slides from film clips) was administered to all groups. Participants then either completed the visuospatial condition Tetris [30], a verbal/conceptual condition Pub Quiz [31] or sat quietly (no-task control condition) for 10 min. During these 10 min, frequency of initial visual intrusions was recorded. Participants had significantly fewer intrusions whilst playing Tetris compared to the no-task condition with no comparable difference between the Pub Quiz game and no-task condition (Table 3). There was no difference in mood at the end of 10 min between the 3 conditions and task compliance was rated equivalently across conditions (Table 4). Participants in the two computer game conditions experienced comparable levels of enjoyment and difficulty for the tasks they performed (Table 4).

**Table 3:** Experiment 1 frequency of initial intrusions during the 10-min experimental manipulation [i.e. during computer game play or no-task condition].

	No-task (n = 20)		Tetris (n = 20)		Pub Quiz (n = 20)		ANOVA
	mean	sem	mean	sem	mean	sem	
Frequency of initial intrusions	12.35	3.40	4.30	1.10	5.90	1.38	*F*_(2,57) = 3.69_ §

§ Tetris relative to no-task control p<0.05; Pub Quiz relative to no-task control = NS.

**Table 4:** Experiment 1 means and statistics for mood assessment, task compliance, game enjoyment and difficulty for the 10-min experimental manipulation which did not differ between conditions.

Measure	No-task (n = 20)		Tetris (n = 20)		Pub Quiz (n = 20)		ANOVA/t-test
	mean	sem	mean	sem	mean	sem	
Post-condition mood	5.81	1.08	4.23	0.79	3.86	0.92	*F*_(2,57)_ = 1.19 (NS)
Task compliance	8.78	0.20	8.45	0.36	8.89	0.24	*F*_(2,57)_ = 0.48 (NS)
Game enjoyment	-	-	7.36	0.33	7.18	0.32	t_(38)_ = 0.37 (NS)
Game difficulty	-	-	3.68	0.49	3.69	0.43	t_(38)_ = 0.01(NS)

**Figure 2:**
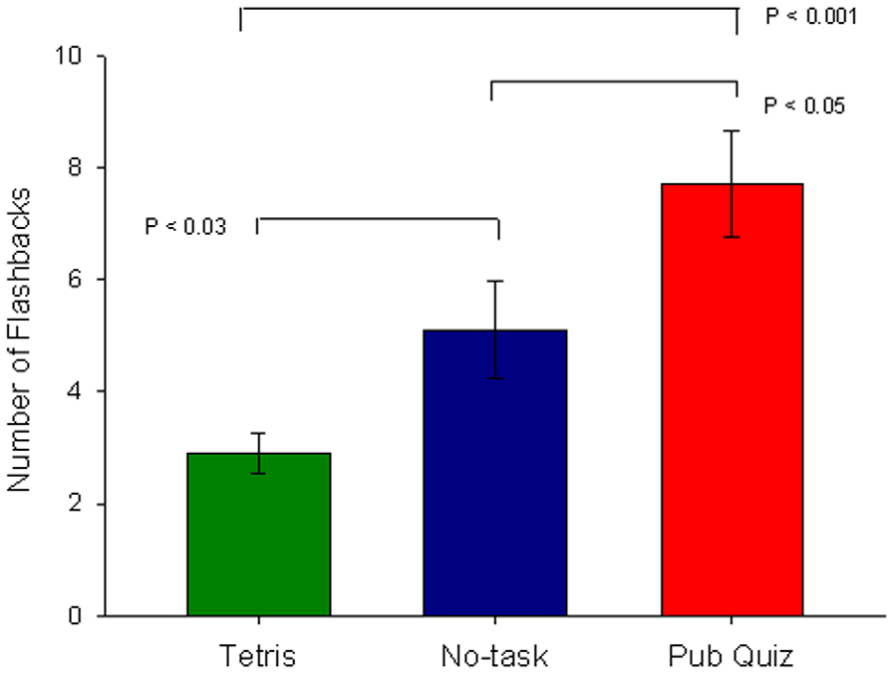
Experiment 1 key outcome variable: flashback frequency in diary over 1-week for the three conditions (mean +/− sem).

After leaving the laboratory, participants then kept a daily structured diary in which they recorded their flashbacks (involuntary visual mental images) to the trauma film over a period of 1 week. Crucially, we found that participants in the visuospatial condition experienced significantly fewer flashbacks over the week than both the no-task (*d* = .70) and Pub Quiz (*d* = 1.21) conditions. Furthermore, participants in the Pub Quiz condition experienced a significantly greater number of flashbacks over the week compared to no-task (*d* = .62) (Figure 2). Diary compliance was equivalent across conditions (Table 5).

**Table 5:** Experiment 1 diary compliance and recognition memory after 1-week indicating equivalence across conditions.

Measure	No-task (n = 20)		Tetris (n = 20)		Pub Quiz (n = 20)		ANOVA
	mean	sem	mean	sem	mean	sem	
Diary compliance	8.60	0.94	8.80	1.32	8.75	0.85	*F*_(2,57)_ = 0.82 (NS)
Recognition memory score	20.95	1.59	20.65	0.64	19.65	1.04	*F*_(2, 57)_ = 0.34 (NS)

On a recognition memory test for the trauma film given after 1 week, performance was comparable in the Tetris, Pub Quiz and no-task conditions (Table 5), indicating that voluntary memory for the film was not differentially impaired. That is, completing the computer game tasks had served only modulate involuntary trauma flashbacks typically associated with psychological distress and dysfunction, but not the actual deliberately recalled memories for the events when asked to remember them.

### Experiment 2

75 non-clinical participants took part in Experiment 2 with no baseline differences between the three conditions (Table 6). There was no baseline difference in mood pre-film, and mood deterioration was equivalent across conditions post-film (Table 7).

**Table 6:** Experiment 2 means and statistics for age and baseline assessments indicating experimental conditions were equivalent at baseline.

Measure	No-task (n = 26)		Tetris (n = 26)		Pub Quiz (n = 26)		ANOVA
	mean	sem	mean	sem	mean	sem	
Age	24.62	1.89	22.04	1.03	22.23	1.11	*F*_(2,75)_ = 1.05 (NS)
Beck Depression Inventory (BDI)	6.23	1.21	6.12	1.08	5.65	4.28	*F*_(2,75)_ = 0.83 (NS)
Trait Anxiety (STAI-T)	36.58	2.12	36.81	1.78	37.58	1.60	*F*_(2,75)_ = 0.08 (NS)
Traumatic Experiences Questionnaire (TEQ)	1.46	0.32	0.92	0.33	1.08	0.25	*F*_(2,75)_ = 0.84 (NS)

**Table 7:** Experiment 2 means and statistics for mood assessment (pre vs. post-trauma film) indicating equivalent deterioration in mood over the film across conditions.

	No-task (n = 26)		Tetris (n = 26)		Pub Quiz (n = 26)		ANOVA		
	mean	sem	mean	sem	mean	sem	Time	Group	Group* Time
Pre-film mood	3.09	0.77	4.04	0.67	3.49	0.68	*F*_(2, 75)_ = 64.27§	*F*_(2, 75)_ = 0.63(NS)	*F*_(2, 75)_ = 0.15(NS)
Post-film mood	7.05	1.11	8.52	1.01	7.31	1.03			

§ p<0.01.

**Figure 3:**
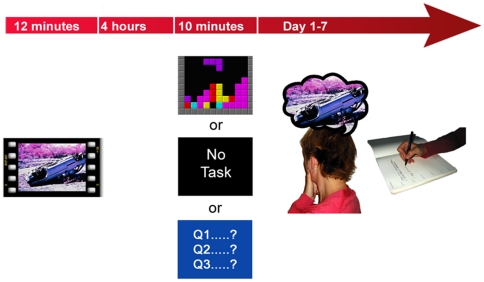
Experiment 2 study design overview. Participants completed the trauma film paradigm. All participants viewed a traumatic film followed by a 4-hr break where participants went about their daily business. Participants then returned to the laboratory and were allocated to one of three experimental conditions (as in experiment 1) which they completed for 10 min. Participants in the computer game conditions rated their enjoyment of the game. Flashbacks (involuntary memories) were monitored for 1 week using an intrusion diary. After 1 week, diary content and compliance was checked and a test of voluntary memory (recognition memory test) for the trauma film was administered.

After the film participants left the laboratory for 4 hours and were free to go about their daily business during this time. Upon return, intervention procedures were identical to that of the three conditions in Experiment 1 (Figure 3). The frequency of initial intrusions participants experienced during the 10-min experimental intervention was reduced in both the Tetris and Pub Quiz conditions relative to the no-task condition (Table 8). After the 10-min intervention, mood and task compliance were comparable across conditions (Table 9). Participants in both computer game conditions experienced comparable levels of enjoyment and difficulty (Table 9).

**Table 8:** Experiment 2 frequency of initial intrusions during the 10-min experimental manipulation [i.e. during computer game play or no-task condition].

	No-task (n = 26)		Tetris (n = 26)		Pub Quiz (n = 26)		ANOVA
	mean	sem	mean	sem	mean	sem	
Frequency of initial intrusions	9.84	1.31	5.65	0.94	6.00	0.67	*F*_(2, 75)_ = 5.25§

§ Tetris relative to no-task control p<0.05; Pub Quiz relative to no-task control p<0.05.

**Table 9:** Experiment 2 means and statistics for mood assessment, task compliance, game enjoyment and difficulty for the 10-min experimental manipulation which did not differ between conditions.

Measure	No-task (n = 26)		Tetris (n = 26)		Pub Quiz (n = 26)		ANOVA/t-test
	mean	sem	mean	sem	mean	sem	
Post condition mood	4.76	1.22	3.78	0.65	4.62	0.87	*F*_(2,75)_ = 0.73 (NS)
Task compliance	8.50	0.38	8.51	0.26	8.71	0.35	*F*_(2,75)_ = 0.88 (NS)
Game enjoyment	-	-	6.60	0.28	6.46	0.28	t_(50)_ = 0.33 (NS)
Game difficulty	-	-	2.89	0.35	3.69	0.32	t_(50)_ = 0.10 (NS)

**Figure 4:**
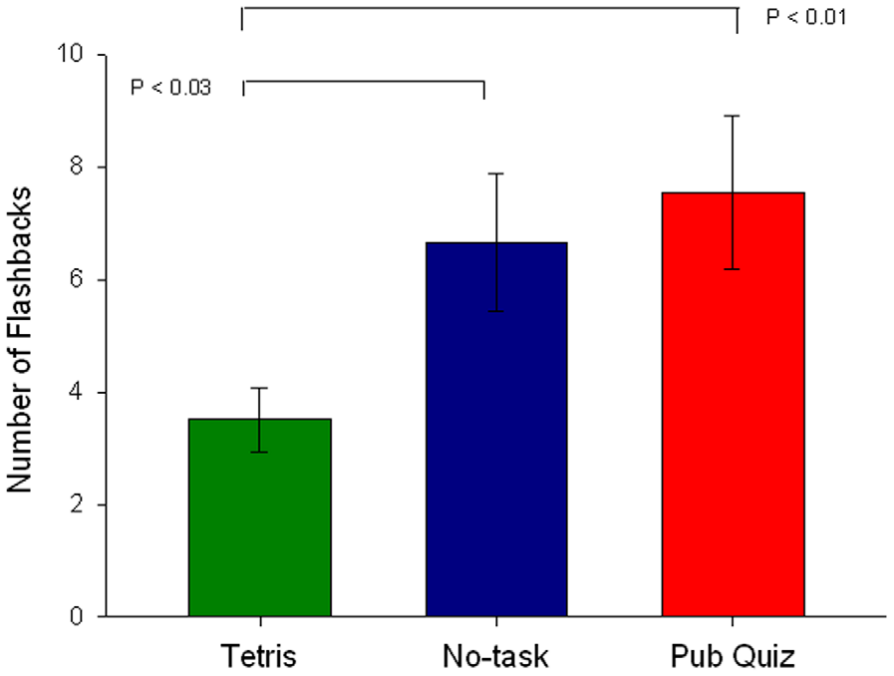
Experiment 2 key outcome variable: flashback frequency in diary over 1-week for the three conditions (mean +/− sem).

Participants' week long diaries recording flashbacks to the trauma film show that those in the Tetris condition experienced significantly fewer flashbacks over the week than the no-task control condition (*d* = .62) (Figure 4). Relative to Tetris, there were significantly more flashbacks after Pub Quiz (*d* = .70). The relative increase in flashbacks after Pub Quiz condition compared to the control condition was not significant (Figure 4). Diary compliance was comparable across conditions, as was the recognition memory test at one week (Table 10).

**Table 10:** Experiment 2 diary compliance and recognition memory after 1-week indicating equivalence across conditions.

Measure	No-task (n = 26)		Tetris (n = 26)		Pub Quiz (n = 26)		ANOVA
	mean	sem	mean	sem	mean	sem	
Diary compliance	8.53	0.24	8.46	0.23	8.53	0.19	*F*_(2,75)_ = 0.96 (NS)
Recognition memory score	20.48	0.50	19.25	0.64	20.03	0.52	*F*_(2,75)_ = 1.32 (NS)

Cohen's d is a standardized measure of effect size in which values between 0.2 to 0.3 are taken to indicate “small” effect, values around 0.5 indicate a “medium” effect and values >0.8 indicate a “large” effect.

## Discussion

We have proposed that engaging in a visuospatial task such as Tetris may offer a ‘cognitive vaccine’ [1] against the development of PTSD flashbacks after exposure to traumatic events. Flashbacks are the hallmark feature of PTSD, thus offering an important target for intervention. At present, preventative interventions for PTSD remain lacking. Following our recent finding that playing Tetris post-trauma compared to no-task may reduce the development of flashbacks [1], the current experiments provide important steps in translating this intervention for clinical real-world application.

First, the current experiments provide the first evidence that different computer games have differential effects on the development of flashbacks post-trauma. Playing Tetris after viewing traumatic material reduced later flashbacks compared to no-task control (Experiment 1 and 2), whereas the computer game Pub Quiz did not. In fact, Pub Quiz even worsened these post-trauma symptoms in Experiment 1. This was the case despite the games being rated as equally as enjoyable and as of similar difficulty. There is increasing interest in using computer games as novel psychological interventions in healthcare [32] but our data suggest that in the field of trauma, not all computer games are beneficial or even merely distracting - some may even be harmful. We account for this differential pattern of findings via our model of trauma memory formation from cognitive science, as outlined in the Introduction. We note that there is a lively debate about the modality specificity of working memory [20], [33], [34], [35], [36], [37], [38], [39], [40], [41] and that the current data is supportive in differentiating visual versus verbal aspects as well as holding clinical utility.

Second, our data are the first to demonstrate that the beneficial effects of Tetris on later intrusive memories are retained even when played at 4 hours post-trauma. We account for this based on current models of memory consolidation indicating that certain types of memory may be malleable for up to 6 hours [21]. Clinically, this is an important extension to our previous findings [1] since it considerably extends the time window within which such an intervention might be delivered post-trauma in the real world. We note that in the current paradigm, a reminder of the trauma is given prior to the intervention in order to “reactivate” memories, which is also a feature of paradigms intervening on fear memory reconsolidation [42], [43] and may represent an important clinical step.

### Implications and limitations of findings

The insights arising from these two studies support the possibility that tasks such as Tetris (a simple visuospatial task) may be developed as a post-trauma intervention to reduce the flashback symptoms of PTSD, and administered up to 4 hours post-trauma. Such an approach would provide a novel alternative to drug treatment or counseling. Current clinical treatment reviews suggest that an existing counseling early intervention for trauma - “critical incident stress debriefing” - may worsen rather than improve PTSD symptoms [44], and that there are no drugs yet shown to be effective. There have been recent concerns over the development of certain drugs (e.g. propanolol [45]) to ‘eradicate’ flashback memories after trauma. This issue has been discussed [46], [47] in view of the potential deleterious effects of pharmacological interventions post-trauma on deliberate memory recall. In particular, the implications for court testimony has been a topic of concern [48]. We believe that that rather than “erasing” all trauma memory using pharmacological means, or “suppressing” unwanted memories using cognitive approaches [49], [50], [51], [52], it is important for individuals to be able to choose to remember a traumatic event. We argue that this may be important clinically [53] as well as practically e.g. for legal proceedings. In the current data, deliberate memory recall after playing Tetris, as assessed using a recognition memory task, was left intact. This demonstrates as predicted that playing Tetris only dampens the intrusive, involuntary feature of flashback memories while deliberately remembered knowledge about the event remains.

The current data also suggest that some computer games, such as the verbal game Pub Quiz, are unlikely to have a beneficial effect post-trauma, and may even have harmful effects on flashbacks when played soon after. A further implication of our findings is that the seemingly simple, innocuous activities people may choose to engage in after trauma - such as playing different types of computer games - may differentially affect their subsequent mental health. The increase in flashbacks when Pub Quiz is played at 30 min suggests is consistent with our account of nature of trauma memory whereby verbal/conceptual interference may worsen flashbacks in the consolidation phase, as outlined in the Introduction. It is possible that this effect diminishes over the passage of time (Experiment 2) although further research is needed to study further time points. It would also be useful to explore convergent measures of flashback reporting to the existing self-report diary methodology (e.g. via PDA's or mobile phone SMS technology; see [54] for a review). Our main focus has been on the impact on involuntary (flashback) memory, however further research is also warranted to explore deliberate (voluntary) memory through refined recognition memory tasks including visual elements. Overall, the findings support established models of trauma memory processing [24], [25], [26], [55]. However, our current methodology does not allow any firm conclusions to be drawn regarding the precise mechanisms involved (e.g. the role of retroactive interference [56], [57]) and this remains to be further explored. However, the possibility of modulating aspects of trauma memory post-event is consistent with exciting work emerging in cognitive neuroscience about modulating fear memory during reconsolidation (e.g. [42], [43]), applied to the first time (to our knowledge) in the current line of enquiry to intrusive aspects of memory. Critical next steps will be to further delineate effective mechanisms and seek to test application after a real traumatic rather than film stimulus.

In conclusion, our approach follows directly from hypothesis-driven theory from cognitive science of trauma memory and the visual imagery nature of flashbacks. Data show a differential impact of two types of cognitive task on the development of flashbacks. Thus by directly comparing two computer games and extending the intervention time window, these findings present critical steps required for the development of a computerized ‘cognitive vaccine’ against the development of flashbacks for trauma.

## Materials and Methods

### Experiment 1

Approval was obtained from the University of Oxford Central University Research Ethics Committee. Healthy volunteers were recruited using advertisements placed online and in the local community. Similar to previous studies [1], [17], [22], [61], participants were not permitted to participate if they had received treatment for mental health problems. Sixty participants (aged 18–60 years; mean age = 27 years; 30 females) provide written informed consent and completed baseline assessments of trauma history, mood, trait anxiety and depression. Trauma history was assessed using the Traumatic Experiences Questionnaire, a 12-item check list adapted from criterion A list of the Posttraumatic Diagnostic Scale [PDS; 58]. Participants indicated whether they had experienced or witnessed each of the trauma events listed (“yes” or “no” response). “Yes” scores were summed, and could range from 0 (no traumatic event) to 12 (each and every type of traumatic event experienced or witnessed).

A composite mood score was calculated by summing participants' ratings on three visual analog scales for ‘sadness’ ‘hopelessness’ and ‘depressed’. Visual analog scales for mood were anchored from 0 ‘*not at all*’ to 10 ‘*extremely*’[1]. All participants were given a practice trial of both computer game tasks. Participants then viewed traumatic film footage (Figure 1). The 21-min film [previously used in 22] contained 15 clips of traumatic content including fatal road traffic accidents and graphic scenes of human surgery. Following the film, mood assessments were repeated and standardised filler tasks were completed for 30 min (e.g. rating excerpts of classical music for pleasantness and answering simple questions from an encyclopedia) [1].

After the break, all participants were shown a brief film reminder task in which one neutral but recognizable static image from each of the 15 film clips was presented (via slides in PowerPoint) in order to “reactivate” memories for the trauma film [1], [59], [60]. Then according to random allocation to one of three 10-min conditions, participants either completed the visuospatial condition, verbal/conceptual condition or were in a no-task control condition. Participants in the visuospatial condition played the game Tetris [30] on a computer and used the cursor keys to move and rotate falling blocks to complete the largest number of complete rows across the screen. Participants in the verbal/conceptual condition played the general knowledge game Pub Quiz [31] on a computer using the right mouse button to select one correct answer out of four choices in order to get the highest score possible. Pub Quiz questions varied in content to include history, sport, geography, food and drink etc, and were unrelated to the trauma film content (e.g., “With what item of clothing would you associate the word Panama? A = scarf, B = gloves, C = hat, D = coat” and “How many sides has a rhombus? A = six, B = seven, C = four D = five”). In the no-task control condition, participants were asked to sit quietly for 10 min.

During the 10-min experimental task manipulation (above) participants in all conditions recorded the frequency of initial intrusions of the trauma film. Afterwards participants completed mood assessments and rated to what extent they had followed instructions (task compliance) on a visual analog scale anchored from 0 ‘*not at all*’ to 10 ‘*extremely*’. Participants in the computer game conditions were asked to rate how enjoyable (anchored from 0 ‘*not at all*’ to 10 ‘*extremely*’) and how difficult they found playing the game (anchored from 0 ‘*not at all difficult*’ to 10 ‘*extremely difficult*’).

Participants then kept a daily diary for 1 week, in which they recorded the occurrence (frequency) of their flashbacks and then briefly described the content of each of their flashbacks separately (for verification) e.g. a human knee with blood. Flashbacks backs were described as spontaneously occurring image-based intrusive memories of scenes from the trauma film [based on 1], [17], [22], [61] e.g. the previous flashback of a knee was deemed to be linked to a film scene containing knee surgery. The A5 sized diary was structured in a tabular form with each day broken down into a grid for ‘morning’, ‘afternoon’ and ‘evening’. The frequency data was entered into this grid, and its corresponding content was recorded on lined pages also within the diary. Participants were asked to record all flashbacks immediately after they occurred (whenever possible) and to set aside a regular daily time slots to ensure that their diary was up-to-date. If participants had experienced no flashbacks during any period they were asked to write zero in the diary.

On return to the laboratory 1 week later, the experimenter went through the diary to verify that the content of each of the flashbacks came from a scene in the trauma film watched 1 week earlier (if not, they were discounted). Participants rated the extent to which they had been able to accurately record their flashbacks in the diary (diary compliance) from 1 ‘*not at all accurate*’ to 10 ‘*extremely accurate*’.

Participants completed a recognition memory task as a measure of voluntary memory for the trauma film. The recognition memory task consisted of 30 written statements describing the film (e.g. A policeman stands watching the wreckage whilst making notes on a clipboard) to which they responded ‘true’ or ‘false’. Participants were then debriefed and reimbursed for taking part.

### Experiment 2

The method for Experiment 2 was largely identical to that of Experiment 1 with the main difference being the extension of the time frame from 30 min to 4 hours between watching the film and conducting the experimental task manipulation (Tetris, Pub Quiz or no-task control). In addition a 12-min trauma film [as used in 1] consisting of 11 clips, 7 of which were used in Experiment 1, was viewed.

78 participants (aged 18–57; mean age = 22.9; 42 females) took part in Experiment 2. After post film mood assessments participants left the laboratory, resumed their daily business, and returned 4 hours later. Upon their return participants were shown a brief reminder task in which one recognizable static image from each of the 11 film clips was presented, via slides in PowerPoint, in order to “reactivate” memory for the film. They were then allocated to one of three 10-min conditions where they either played Tetris, Pub Quiz or sat quietly in the no-task condition. As in Experiment 1 participants recorded their initial intrusions during the 10-min experimental task manipulation. Participants then kept a structured diary for 1 week to record flashbacks. Measures of diary compliance and a recognition memory task were taken 1 week later.
