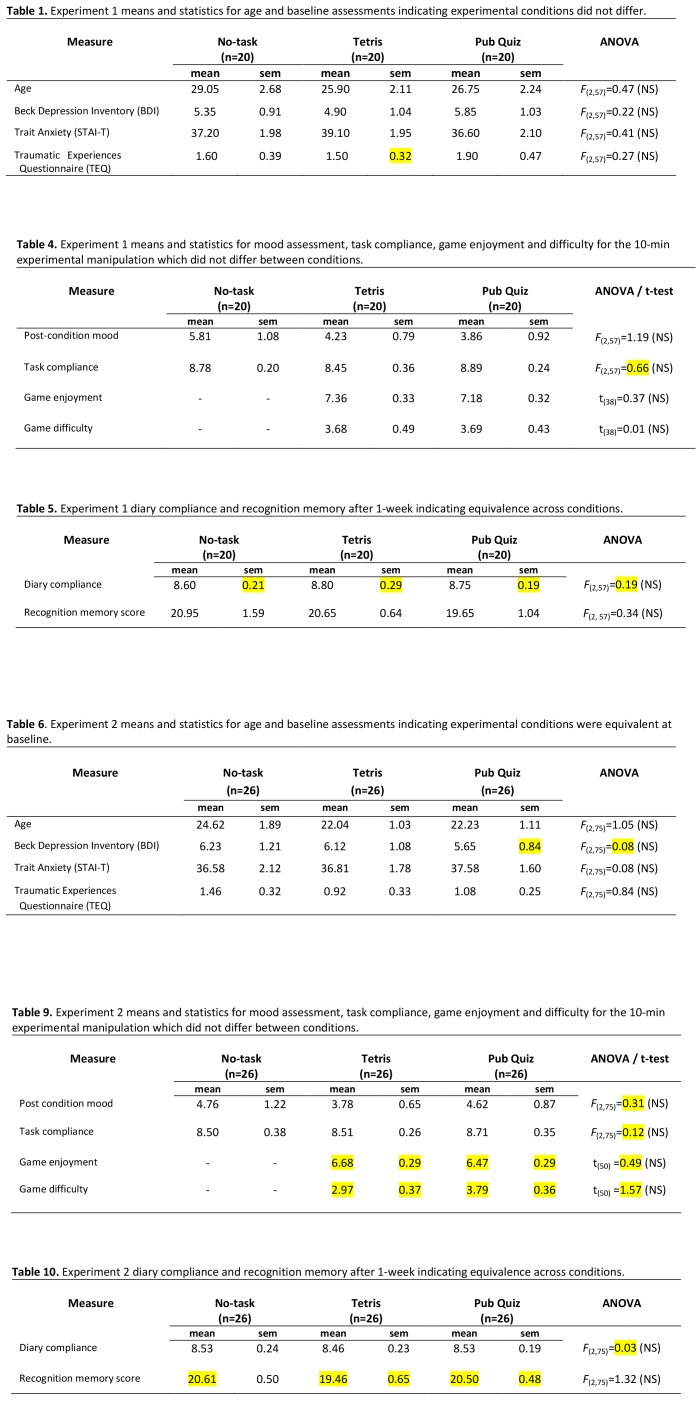# Correction: Key Steps in Developing a Cognitive Vaccine against Traumatic Flashbacks: Visuospatial Tetris versus Verbal Pub Quiz

**DOI:** 10.1371/annotation/eba0a0c8-df20-496b-a184-29e30b8d74d0

**Published:** 2012-11-14

**Authors:** Emily A. Holmes, Ella L. James, Emma J. Kilford, Catherine Deeprose

There were errors in Tables 1, 4, 5, 6, 9, and 10. The pattern of results remained unchanged. The corrected tables can be viewed here: 

**Figure:**